# Effects of Mogroside V on Quality and Antioxidant Activity of Boar Frozen–Thawed Sperm

**DOI:** 10.3390/antiox14060622

**Published:** 2025-05-23

**Authors:** Heming Sui, Xin Wang, Kunlong Hu, Xiaoyu Zuo, Haonan Li, Zhengyu Diao, Jiajing Feng, Yunhai Zhang, Zubing Cao

**Affiliations:** Anhui Province Key Laboratory of Local Livestock and Poultry, Genetical Resource Conservation and Breeding, College of Animal Science and Technology, Anhui Agricultural University, Hefei 230036, China

**Keywords:** antioxidant, frozen–thawed semen, cryopreservation, sperm quality, oxidative stress

## Abstract

Cryopreserved pig semen tends to produce excessive reactive oxygen species (ROS) during the thawing process, which leads to a decline in semen quality during in vitro storage. Mogroside V (MV) has been proven to be an effective antioxidant, and previous research has shown that MV can delay oocyte aging and improve the in vitro maturation efficiency of pig oocytes. However, the role of MV in the cryopreservation capacity of animal sperm remains unclear. To evaluate the effect of MV on sperm motility after thawing, different concentrations of MV (0, 25, 50, 75, 100 μmol/L) were added to the thawing medium. By comparing the sperm motility and kinematic parameters in the thawing medium with different MV concentrations and incubation times (0, 1, 2, and 4 h), we ultimately selected sperm thawed immediately in the medium supplemented with 75 μmol/L MV for subsequent experiments. Compared with the control group, the sperm thawing medium containing MV improved sperm quality during the freeze–thaw process. Immediate evaluation after thawing at 37 °C showed that supplementation with 75 μmol/L MV produced an optimal effect on the maintenance of motility, plasma membrane integrity, the acrosome integrity, the ROS levels, and the T-AOC activity. In conclusion, MV supplementation improves the quality of frozen–thawed sperm by enhancing sperm function and preventing oxidative stress.

## 1. Introduction

The cryopreservation of sperm is an effective method for preserving male fertility. In humans, it is commonly used for patients with low sperm count, those undergoing in vitro fertilization, or individuals seeking to preserve fertility prior to cancer treatment [1,2]. In livestock, this technology has also been widely applied, particularly with cryopreserved bull and boar semen, which plays a crucial role in livestock breeding. In recent years, researchers have been dedicated to improving the quality of frozen boar semen and the success rates of artificial insemination (AI) [3]. The technology for preserving boar semen in vitro is essential for the advancement of AI techniques. However, the proportion of artificial inseminations using frozen boar semen in actual production remains below 1% [4]. There remains a notable gap in AI success between frozen and fresh boar semen, with conception rates for frozen semen ranging from 30% to 60%, compared to up to 90% for fresh semen. Various technical challenges exist with frozen boar semen, and the mechanisms behind cryoinjury may be linked to the cold shock of the sperm and oxidative stress arising from changes in the sperm’s antioxidant defense system [5]. Despite these challenges, the benefits of frozen boar semen technology are substantial. It can reduce the number of boars required for breeding and eliminate temporal and geographical constraints, thereby accelerating breed improvement. Additionally, it effectively controls the spread of diseases, particularly during the African swine fever epidemic, contributing to biosecurity measures.

Studies have shown that an excess of ROS is readily generated during the cryopreservation and thawing of semen [6], which induces oxidative stress in sperm and compromised sperm quality. Extending the in vitro preservation time of boar semen while maintaining the stability of its genetic material is crucial [7]. Additionally, the ability of pig sperm to withstand oxidative stress damage mainly relies on seminal plasma, and its intrinsic antioxidant defense system is limited. As a result, researchers are focused on finding exogenous antioxidants to mitigate the oxidative damage to sperm [8,9]. The addition of these antioxidants to semen diluents and thawing solutions can protect sperm from ROS attacks and enhance the quality of in vitro semen preservation [10,11,12]. For instance, studies have demonstrated that incorporating 50 μmol/L Notoginsenoside R1 as an antioxidant in boar extender improves sperm motility, membrane integrity, and acrosome integrity while reducing ROS levels and lipid peroxidation [13]. Similarly, adding 50 mM resveratrol to cryoprotectants enhances antioxidant enzyme activity and AMPK phosphorylation levels in frozen–thawed boar sperm, while decreasing lipid peroxidation and ROS levels after thawing [14].

MV is the primary active component in the fruit of Siraitia grosvenorii, a cucurbitane-type triterpenoid glycoside with characteristics such as high sweetness, low caloric content, and low toxicity [15,16,17]. The physiological functions of MV are diverse, with reported anti-inflammatory [18,19,20], anti-allergic [21], and anti-cancer [22] activities. Additionally, MV is an effective scavenger of ROS in vitro. Liu et al. discovered that mogroside extracts exhibit strong inhibitory effects on ROS, highlighting their potential as novel anti-glycation agents for reducing blood glucose levels [23,24]. MV has also been shown to reduce fat accumulation in mouse livers and increase phosphorylated AMP-activated protein kinase levels, indicating its role in influencing obesity and non-alcoholic fatty liver disease through enhanced fat metabolism and antioxidant defenses [25]. While the antioxidant activity of MV across multiple tissues is well-established, few studies have examined its protective effects on mammalian germ cells. Junyu Nie and colleagues demonstrated that the addition of MV to pig embryo culture can reverse cytoskeletal abnormalities, mitochondrial dysfunction, and early apoptosis in aged oocytes by upregulating SIRT1 expression [26]. Furthermore, Nie et al. confirmed that MV enhances mitochondrial function and scavenges cytoplasmic ROS when added to the in vitro maturation medium of pig oocytes, thereby improving the developmental competence of parthenogenetic embryos [27]. In another study, Ke Peng et al. found that MV effectively protects pig oocytes from heat stress (HS), likely through the inhibition of HS-induced oxidative stress [28]. However, the protective effects of MV on boar sperm during the freeze–thaw process remain underexplored. The Huoshou black boar, a native pig breed of Anhui Province, represents a significant genetic resource in China. This study employed semen from purebred Huoshou black boars as a research model to examine the protective effects of MV on cryopreserved sperm. Specifically, the research aimed to evaluate the impact of MV on post-thaw sperm quality, including motility, kinetic parameters, abnormality rate, DNA integrity, and other associated metrics. Additionally, this study sought to investigate the potential role of MV in protecting sperm from oxidative stress induced by ROS, thereby enhancing its overall functionality post-thaw.

## 2. Materials and Methods

Reagents used in this study were purchased from Sigma (SigmaAldrich, St Louis, MO, USA) unless otherwise stated. All experiments using boars were conducted in accordance with the Institutional Animal Care and Use Committee (IACUC) guidelines under current approved protocols at Anhui Agricultural University.

### 2.1. Chemicals Preparation

The purchased MV (BP0019, Chengdu Purefa Technology Development Company Limited, Chengdu, China) with 99% purity was dissolved in pure water to obtain a 1 mM stock solution. One gram of frozen semen diluent (Green Auris, Beijing, China) was fully dissolved in 20 mL of pure water and preheated to 37 °C for later use (the exact composition of the commercial reagents is proprietary information and has not been disclosed by the manufacturer). The thawing solution for the control group was the basic thawing solution without MV supplementation. The samples of the control groups and the treatment groups were repeated at least three times.

### 2.2. Animals, Semen Collection, and Sample Preparation

Twelve sexually mature Huoshou black boars were raised under the same management conditions, with controlled environmental temperature, identical diets, 16 h of combined natural and artificial light exposure, and free access to drinking water. Sperm-rich fractions were collected using the gloved-hand method, where a trained technician wearing sterile latex gloves manually stimulated the boar’s penis, gently grasped the spiral glans upon erection, and collected the sperm-rich fraction into a pre-warmed (37 °C) sterile container after discarding the initial 10–15 mL (sperm-poor fraction). Semen was collected twice per week from each boar and three ejaculates per boar were used for sperm freezing. Fresh semen was weighed using an electronic scale, and motility and density were assessed with a sperm analyzer. Throughout this study, ‘semen’ refers to the complete ejaculate containing both spermatozoa and seminal plasma, while ‘sperm’ specifically denotes spermatozoa after separation from seminal plasma. Only semen of high quality, milky white in color, without any foreign matter or odor, and with motility greater than 80% was selected for freezing. The freezing procedure was conducted in two steps. First, the qualified semen (whole ejaculate) was proportionally diluted with pre-dilution solution and placed in a 17 °C refrigerator for initial cooling for about 4 h. After reaching equilibrium at 17 °C, the semen was centrifuged at 800× *g* for 15 min in a large-capacity centrifuge. The supernatant (seminal plasma) was removed, and the sperm pellet (spermatozoa concentrate) was immediately re-diluted with a commercial freezing medium (Green Auris, Beijing, China). This diluted sample was placed in a 17 °C water bath cup prepared in advance, and then transferred to a 4 °C refrigerator for the second cooling for approximately 4 h. Once equilibrated to 4 °C, the semen was further diluted with an isothermal cryoprotective solution (Green Auris, Beijing, China) to an equal volume. The diluted semen was then filled into 0.5 mL frozen fine tubes (Minitube, Munich, Germany) and sealed with the sealing powder. The semen was frozen and stored in liquid nitrogen for at least two months prior to use in research.

### 2.3. Experimental Protocols

The experiment included 5 groups (0, 25, 50, 75, and 100 μmol/L MV, 3 repetitions per group). Each prepared thawing solution was preheated in a 37 °C water bath for 10 min before use for thawing frozen semen. The frozen fine tubes were retrieved from the liquid nitrogen and quickly placed in a 50 °C water bath, where they were gently swung for 15 s. Sterile absorbent paper was used to dry the surface of the tubes. Next, the sealed end of each tube was cut, and the contents were expelled into the thawing solution. The semen was mixed well and set aside for further testing.

### 2.4. Detection of Sperm Motility and Kinetic Parameters

Frozen semen thawed in a solution without MV served as the control group, while thawing solutions containing MV at concentrations of 25, 50, 75 and 100 μmol/L were used for the experimental groups. The motility and kinetic parameters of frozen–thawed sperm were measured after incubation at 37 °C for 0, 1, 2, and 4 h to identify the optimal MV concentration. Sperm motility and kinetics parameters were evaluated by computer-assisted sperm analysis system (ZhuXianzi, Guangzhou, China), which consisted of a trinocular phase-contrast microscope, heating area, and digital camera. A total of six sperm dynamic parameters were measured for each sample by analyzing five randomly selected regions (each containing at least 200 sperm): average path velocity (VAP, μm/s), straightness (STR, %), wobble (WOB, %), beat cross frequency (BCF, Hz), curvilinear velocity (VCL, μm/s), and straight-line velocity (VSL, μm/s). The standard parameter settings were as follows: frames per second: 60; minimum of frames acquired: 21; and sperm with VAP ≥ 10 μm/s were classified as motile.

### 2.5. Measurement of Sperm Survival Time

Following semen thawing, the sperm density was adjusted to 1 × 10^6^ cells/mL and incubated at 37 °C in a 5% CO_2_ environment, with timing beginning at incubation onset. After 30 min, the sample was retrieved and centrifuged at 1600× *g* for 1 min, and sperm density was re-adjusted to 1 × 10^6^ cells/mL. The sample was then incubated under the same conditions for periodic viability detection. Timing ended when sperm motility dropped below 1%, with the recorded time in minutes representing the sperm survival duration [29].

### 2.6. Evaluation of Sperm Morphology

Sperm morphological abnormalities were detected by eosin staining (E8090, Solarbio, Beijing, China). The types of abnormal sperm mainly include three categories. The first category is head defects, such as large head, small head, conical head, pear-shaped head, double head, etc. The second category is neck and midpiece defects, such as neck curvature, thick and irregular midpiece, abnormally thin midpiece, etc. The third category is tail defects, such as short tail, multiple tails, tail breakage, etc. The sperm density was adjusted to 2 × 10^4^ cells/mL, and a 10 μL semen sample was placed on a slide. A smear was prepared using a coverslip and allowed to air-dry at room temperature. Next, the dried smear was immersed in methanol for fixation. After 10 min, the smear was retrieved and air-dried once more. Subsequently, the smear was stained with a 2% eosin staining solution for 15 min at room temperature, then gently rinsed with pure water on both sides. Finally, sperm morphology and abnormality rates were observed and quantified under a light microscope at 400× magnification.

### 2.7. Analysis of Plasma Membrane Integrity

The plasma membrane (PM) integrity of sperm was assessed by the hypoosmotic swelling test (HOST) [30]. The sperm density was adjusted to 1 × 10^6^ cells/mL, and 1 mL of thawed semen was incubated with 9 mL of hypoosmotic solution (7.35 g of sodium citrate and 13.51 g of fructose were dissolved in 1 L of distilled water) at 37 °C for 30 min. To minimize potential membrane damage from sedimentation, the centrifuge tube was gently shaken every 5 min during incubation. Following incubation, 10 μL of the hypoosmotically treated semen sample was dropped on the slide and covered with a coverslip. Sperm tail morphology was then observed under a 400× light microscope (Olympus, Tokyo, Japan). The plasma membrane integrity rate was calculated based on the percentage of sperm with a curled tail among 200 spermatozoa in at least three distinct microscopic fields.

### 2.8. Analysis of Acrosome Integrity

Acrosome integrity in sperm was examined using the isothiocyanate-labeled peanut agglutinin (PNA-FITC) staining method (Sigma, L7381). The sperm density was adjusted to 1 × 10^6^ cells/mL, and 20 μL of the semen sample was spread evenly on a slide and naturally air-dried. The dried slide sample was fixed by immersion in anhydrous methanol for 10 min, then allowed to air dry at room temperature. Next, 200 μL of PNA-FITC staining solution at a working concentration of 0.1 g/mL was evenly added to the slide, which was then incubated in a dark environment at 37 °C under 5% CO_2_ for 30 min. After incubation, the slide was gently rinsed three times with phosphate-buffered saline (PBS) and air-dried. It was then covered with a coverslip and sealed with colorless nail polish. The sample was examined using a fluorescence microscope (Olympus, Tokyo, Japan) at 400× magnification, with excitation and emission wavelengths of 480 nm and 530 nm respectively. At least 200 sperm cells in three separate microscopic fields were counted. The fluorescein-labeled peanut agglutinin dye can bind to the β-D-galactosyl residue on the acrosomal outer membrane glycoproteins, indicating the integrity of the acrosome. Sperm with intact acrosomes exhibited cap-like green fluorescence on their heads, while those with damaged acrosomes showed incomplete or absent fluorescence. The proportion of sperm with intact acrosomes was calculated to determine acrosome integrity.

### 2.9. Analysis of DNA Integrity

Sperm DNA integrity was assessed using acridine orange staining (S19094, Shanghai yuanye Bio-Technology Company Limited, Shanghai, China). The sperm density was adjusted to 2 × 10^4^ cells/mL, and 10 μL of the semen sample was evenly spread on a slide and allowed to air-dry naturally. The sample was fixed in anhydrous ethanol–glacial acetic acid (3:1) for 5 min and then stained with freshly prepared acridine orange solution for 10 min. Following staining, the slide was rinsed with distilled water and allowed to air-dry at room temperature. The sample was examined under a fluorescence microscope (Olympus, Tokyo, Japan) at 400× magnification, with excitation and emission wavelengths of 480 nm and 530 nm, respectively. At least 200 sperm cells from three different microscopic fields were analyzed. Sperm with intact DNA exhibited green fluorescence in their heads, and the proportion of sperm with intact DNA was calculated to determine the DNA integrity rate.

### 2.10. ROS Content Assay

ROS levels of spermatozoa were measured using a reactive oxygen species detection kit (S0033S, Shanghai Beyotime Biotechnology, Shanghai, China). The sperm density was adjusted to 1 × 10^6^ cells/mL, and approximately 10 μL diluted DCFH-DA staining solution was added to spermatozoa, followed by incubation at 37 °C for 15 min. Fluorescence intensity was measured using a microplate reader, with excitation set at 488 nm and emission at 525 nm to obtain the OD values for each well. The fluorescence intensity of each well was recorded and analyzed to compare ROS levels between the control and experimental groups.

### 2.11. MDA Content Assay

The malondialdehyde (MDA) content of spermatozoa was measured using an MDA content test kit (Nanjing Jiancheng Bioengineering Institute, Nanjing, China). Thawed frozen semen was diluted 10-fold, and 2 mL of the diluted sample was placed in a centrifuge tube and centrifuged at 1000× *g* for 5 min to remove the supernatant. Next, 2 mL of ice-cold PBS was added, and the spermatozoa were fully lysed using ultrasound to release the MDA. The sample was then centrifuged at 12,000 rpm and 4 °C for 5 min, and the supernatant was collected for further use. Following the MDA assay kit instructions, the MDA content was measured by mixing the sample with the detection reagent and incubating it in a 98 °C water bath for 45 min. After cooling in an ice bath, the mixture was centrifuged at 12,000× *g* for 10 min. The supernatant was then transferred to a 96-well plate, and absorbance changes were monitored at 532 nm using a fluorescent enzyme-labelling instrument. The measurement of MDA levels typically reflects the degree of lipid peroxidation within the organism, thereby indirectly reflecting the extent of cellular damage.

### 2.12. T-AOC Activity Assay

The total antioxidant capacity (T-AOC) of spermatozoa was measured using a T-AOC test kit (Nanjing Jiancheng Bioengineering Institute, Nanjing, China). The preparation of sperm samples was consistent with the prior determination of MDA content, and the supernatant obtained after ultrasound lysis and centrifugation was retained. Following the manufacturer’s instructions, T-AOC activity was measured by mixing the sample with the detection reagent and allowing it to react at room temperature for 6 min. The absorbance changes at 405 nm were monitored using a fluorescent enzyme-labeling instrument. When Trolox was used as a standard for measuring T-AOC, the antioxidant capacity of the sample was expressed as Trolox-Equivalent Antioxidant Capacity (TEAC). The stronger the total antioxidant capacity, the larger the TEAC value.

### 2.13. Statistical Analysis

All experiments were conducted with three valid biological replicates, and statistical analyses were performed using one-way ANOVA (with Tukey’s post hoc test for multiple comparisons) or Student’s *t*-test (GraphPad Prism 5.0, GraphPad Software, San Diego, CA, USA). Unless otherwise noted, all data were presented as mean ± standard error (mean ± S.E.M), and *p* < 0.05 was considered to be statistically significant.

## 3. Results

### 3.1. Effects of MV on Motility and Kinetic Parameters of Frozen–Thawed Sperm

**Figure 1:**
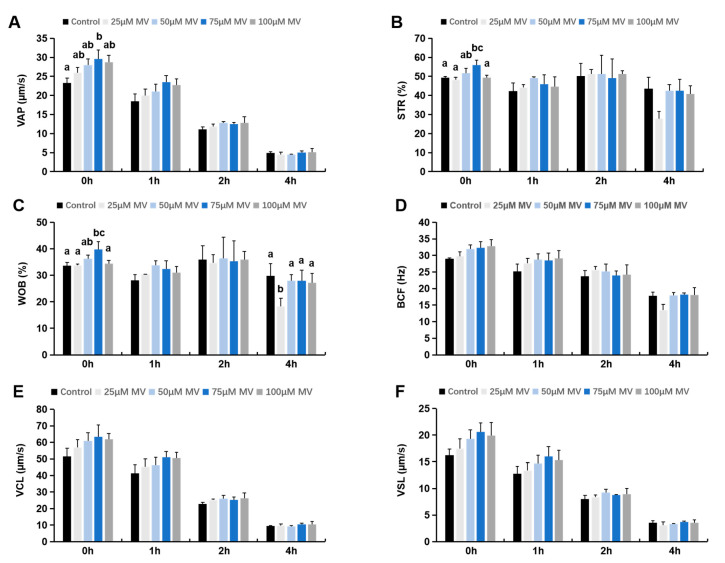
Effects of different concentrations of MV on frozen–thawed sperm motility and kinetic parameters of Huoshou black boar. (**A**) Average path velocity of frozen–thawed sperm of Huoshou black boar. (**B**) Straightness of frozen–thawed sperm of Huoshou black boar. (**C**) Frozen–thawed sperm wobble of Huoshou black boar. (**D**) The beat cross frequency of frozen–thawed sperm of Huoshou black boar. (**E**) Curvilinear velocity of frozen–thawed sperm of Huoshou black boar. (**F**) Straight-line velocity of frozen–thawed sperm of Huoshou black boar. Note: the results were subjected to one-way ANOVA analysis from at least three independent experiments, and all data were presented as mean ± S.E.M. The bars represent the standard error. Different letters (a, b, c) indicate significant differences across groups (*p* < 0.05).

As shown in Table 1, sperm motility was significantly higher in the 75 μmol/L MV treatment group immediately tested after thawing compared to the control (Control: 48.00 ± 2.95, 75 μmol/L MV: 58.20 ± 2.63, *p* < 0.05), while the 25, 50, and 100 μmol/L MV groups showed no significant differences from the control. After 1 h of incubation at 37 °C, the 50, 75, and 100 μmol/L MV groups exhibited significantly higher sperm motility than the control, with 75 μmol/L MV having the most pronounced effect (Control: 40.33 ± 2.28, 50 μmol/L MV: 47.07 ± 0.91, 75 μmol/L MV: 49.27 ± 2.09, 100 μmol/L MV: 47.47 ± 2.14, *p* < 0.05). After 2 and 4 h of incubation at 37 °C, there were no significant differences in motility across MV concentrations and the control group. The effects of adding different concentrations of MV to frozen semen thawed solution on the kinematic parameters of frozen–thawed sperm are displayed in Figure 1. Testing immediately after thawing at 37 °C revealed that the average path velocity (Control: 23.29 ± 1.30, 75 μmol/L MV: 29.56 ± 2.36, *p* < 0.05), straightness (Control: 49.25 ± 0.85, 75 μmol/L MV: 55.91 ± 2.71, *p* < 0.05), and wobble (Control: 33.69 ± 1.24, 75 μmol/L MV: 39.82 ± 2.90, *p* < 0.05) were significantly higher in the 75 μmol/L MV group compared to the control (Figure 1A–C). Meanwhile, the 25, 50, and 100 μmol/L MV groups showed no significant difference from the control. Across all incubation times after thawing at 37 °C, MV addition did not significantly influence other motility parameters, such as sperm beat cross frequency (BCF), curvilinear velocity (VCL), and straight-line velocity (VSL) (Figure 1D–F). In conclusion, 75 μmol/L MV appears to be the optimal concentration for inclusion in the thawing solution. Therefore, 75 μmol/L MV was used in subsequent experiments, with frozen Huoshou black boar semen incubated at 37 °C for 0 h for further analysis.

### 3.2. Effects of MV on Quality Parameters of Frozen–Thawed Sperm

**Figure 2:**
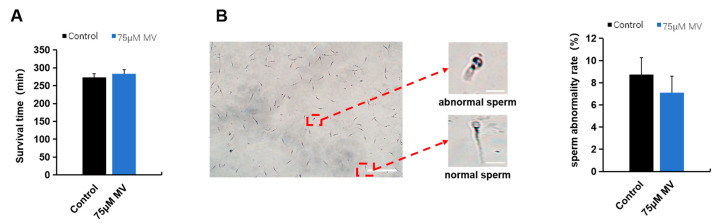
Effects of MV on frozen–thawed sperm quality parameters of Huoshou black boar. (**A**) Survival time of frozen–thawed sperm between control and experimental group. (**B**) Abnormality rate of frozen–thawed sperm between control and experimental group. Scale bar: 50 μm. Data were subjected to Student’s *t*-test from at least three independent experiments, and the results are presented as mean ± S.E.M.

The results of the MV effect on the sperm quality parameters are illustrated in Figure 2. Under incubation at 37 °C, there were no significant differences in sperm survival time and abnormality rate between the 75 μmol/L MV group and the control group (Figure 2A,B).

### 3.3. Effects of MV on Functional Characteristics of Frozen–Thawed Sperm

**Figure 3:**
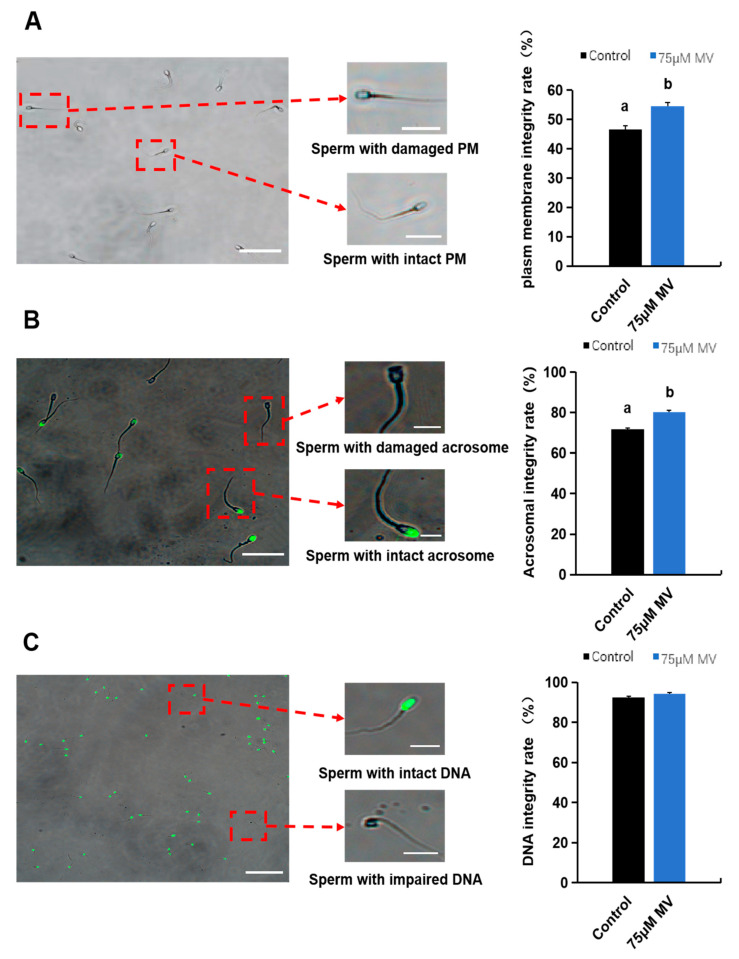
Effects of MV on frozen–thawed sperm functional characteristics of Huoshou black boar. (**A**) Plasma membrane integrity rate of frozen–thawed sperm between control and experimental group. Scale bar: 20 μm. (**B**) Acrosome integrity rate of frozen–thawed sperm between control and experimental group. Scale bar: 20 μm. (**C**) DNA integrity rate of frozen–thawed sperm between control and experimental group. Scale bar: 100 μm. Data were subjected to Student’s *t*-test from at least three independent experiments, and the results are presented as mean ± S.E.M. Different letters (a, b) represent significant differences between the two groups (*p* < 0.05).

The effects of adding MV to frozen semen thawed solution on the functional characteristics of frozen–thawed sperm are presented in Figure 3. The hypoosmotic swelling test was used to evaluate the functional integrity of sperm membranes by observing the morphological changes of sperm in a hypoosmotic environment to assess their functional status. The results showed that the membrane integrity rate of frozen–thawed sperm in the 75 μmol/L MV treatment group was significantly higher than in the control group (Control: 46.52 ± 1.33, 75 μmol/L MV: 54.46 ± 1.37, *p* < 0.05) (Figure 3A). The acrosome and DNA integrity of sperm were assessed via PNA-FITC and acridine orange staining, respectively. The results showed that the acrosome integrity rate of frozen–thawed sperm in the 75 μmol/L MV treatment group was significantly higher than in the control group (Control: 71.74 ± 0.48, 75 μmol/L MV: 80.11 ± 0.87, *p* < 0.05) (Figure 3B). However, there was no significant difference in DNA integrity between the two groups (Figure 3C).

### 3.4. Effects of MV on ROS Content, MDA Content, and T-AOC Ability of Frozen–Thawed Sperm

**Figure 4:**
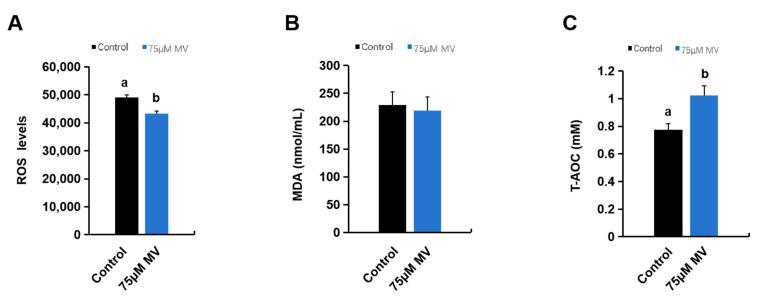
Effects of MV on oxidative stress-related indicators in frozen–thawed sperm of Huoshou black boar. (**A**) ROS level of frozen–thawed sperm between control and experimental group. (**B**) Malondialdehyde content of frozen–thawed sperm between control and experimental group. (**C**) Total antioxidant capacity of frozen–thawed sperm between control and experimental group. Data were subjected to Student’s *t*-test from at least three independent experiments, and the results are presented as mean ± S.E.M. Superscript letters (a, b) indicate statistically significant difference (*p* < 0.05).

ROS are produced by cellular metabolism and can affect sperm function through various mechanisms. We used DCFH-DA fluorescent probes and a microplate reader to quantitatively assess the ROS levels in the control and experimental groups. The results showed that the ROS levels in the frozen–thawed sperm of the 75 μmol/L MV treatment group were significantly lower than those of the control group (Figure 4A). We also measured the effects of MV addition on malondialdehyde (MDA) content and total antioxidant capacity (TAC) in the sperm. The results showed that adding 75 μmol/L MV slightly reduced the MDA content and significantly enhanced the TAC in frozen–thawed sperm (Figure 4B,C).

## 4. Discussion

During the freezing and thawing process, sperm may suffer a series of physical damages, typically manifested as structural injuries and a loss of motility. Therefore, adopting appropriate freezing and thawing methods is essential to maintain sperm quality. The addition of antioxidants derived from herbal plants to the thawing medium can help to mitigate the adverse effects of freezing and thawing [31,32]. These antioxidants not only improve sperm quality but also extend sperm lifespan, enhance fertilization rates, and increase embryo development efficiency [33,34,35]. Mogroside V, a natural herbal extract known for its antioxidant [36,37], antibacterial [38], anti-inflammatory [39,40], and low cytotoxicity [17] properties, has rarely been studied for its protective effects on germ cells in male animals. Based on this, we used purebred Huoshou black boar semen as a research model to investigate the protective effect of MV on cryopreserved semen.

Cryopreservation can induce lipid peroxidation reactions in mammalian sperm. Studies have shown that lipid peroxyl radicals contribute to the production of MDA in boar sperm [41], and research by Jordi Roca and colleagues also confirmed that these radicals may be the primary ROS responsible for cryodamage in boar sperm [42]. In addition to impairing membrane integrity and motility, lipid peroxidation can also damage DNA in the sperm nucleus [43]. In this study, we found that adding an appropriate amount of MV to the thawing solution of frozen boar semen significantly improved sperm motility and certain kinematic parameters. Notably, the addition of 75 μmol/L MV provided the most significant protective effect on the quality of thawed sperm, indicating that MV helps to maintain sperm motility during cryopreservation and enhances recovery during the thawing process. When analyzing the survival time and abnormality rate of sperm between the 75 μmol/L MV group and the control group, no significant differences were observed. Ideally, an effective MV concentration should significantly extend sperm survival time and reduce the abnormality rate. Although no significant differences were observed between the 75 μmol/L MV group and the control group, we can still infer that adding 75 μmol/L MV to the thawing solution might offer potential advantages over the control group. Additionally, we used the hypoosmotic swelling test to assess sperm plasma membrane integrity. If the sperm membrane integrity is intact, specific swelling patterns, such as bent and swollen tails, can be observed. We found that the plasma membrane integrity rate of frozen–thawed sperm in the 75 μmol/L MV treatment group was significantly higher than that of the control group. This indicates that adding 75 μmol/L MV to the thawing solution can effectively reduce membrane damage and protect the plasma membrane integrity of thawed Huoshou black boar sperm. The principle of PNA-FITC staining for detecting sperm acrosome integrity is based on the specific binding of peanut agglutinin (PNA) to β-D-galactosyl residues in the acrosomal region, visualized through fluorescence labeling with fluorescein isothiocyanate (FITC). When the acrosomal structure is intact, PNA-FITC binds uniformly to the outer membrane and contents of the acrosome, appearing as a strong green fluorescence on the sperm head under a fluorescence microscope. Conversely, when the acrosome is damaged or has undergone an acrosomal reaction, the rupture of the acrosomal membrane leads to the exposure or loss of binding sites, resulting in weakened fluorescence signals or fluorescence localized only in certain regions. Our experimental results showed that the acrosome integrity of frozen–thawed sperm in the 75 μmol/L MV treatment group was significantly higher than that in the control group. This indicates that this treatment may provide a protective effect on the acrosomal structure of sperm.

Sperm is the first reported cell type to exhibit potential susceptibility to oxidative stress. Increasing evidence suggests that oxidative stress significantly impacts the quality of cryopreserved boar semen [44]. While some damage caused by oxidants can be repaired, sperm often lack the necessary cytoplasmic enzyme repair systems, preventing recovery from oxidative stress-induced damage [45]. During the freezing and thawing process, semen generates ROS [46]. An appropriate amount of ROS is crucial for processes such as sperm capacitation, hyperactivation, the acrosome reaction, and fusion with the oocyte [47]. However, a prolonged in vitro preservation time of sperm leads to nutrient depletion and a decline in antioxidant enzymes, resulting in the excessive accumulation of free radicals. When the antioxidant defense system in the semen is insufficient to counteract the ROS generated by its metabolism, excess ROS can attack the DNA, proteins, and plasma membrane of sperm cells, causing oxidative damage and inducing oxidative stress. This negatively affects sperm structure, motility, fertilization capacity, and even subsequent embryo development, ultimately resulting in poor breeding performance [48]. Boar sperm has lower antioxidant capacity compared to other species, making it more susceptible to oxidative damage [49]. Our study found that compared to the control group, adding 75 μmol/L MV to the thawing solution significantly reduced ROS levels, indicating that MV can alleviate oxidative damage to frozen sperm during the thawing process. T-AOC represents the cumulative ability of all enzymatic and non-enzymatic antioxidants in a biological system to neutralize ROS, thereby protecting cells from oxidative damage. Our results demonstrate that, compared to the control group, the addition of 75 μmol/L MV effectively enhances the antioxidant capacity of frozen semen, mitigates oxidative stress-induced damage during the freeze–thaw process, and improves the recovery outcomes of cryopreserved sperm.

Our research findings indicate that adding 75 μmol/L MV to the thawing solution of cryopreserved semen is beneficial for improving sperm motility and functionality while reducing oxidative stress. The findings of this study align with previous reports on the antioxidant properties of MV, confirming that MV can serve as a novel exogenous semen additive for the in vitro preservation of Huoshou black boar semen. In fact, previous studies have demonstrated the antioxidant properties of Momordica charantia saponins and cucurbitacin B. Momordica charantia saponins have been shown to lower blood glucose and lipid levels, exerting antioxidant effects that alleviate diabetic symptoms in mice. Cucurbitacin B can inhibit COX-2 and NOS activity, reduce oxidative stress, suppress pro-inflammatory cytokines, and modulate adaptive immune proteins, playing roles in both innate and adaptive immunity [50,51]. Both antioxidants belong to the cucurbitane-type triterpenoid glycosides, the same class as MV. These findings suggest that MV may share similar molecular mechanisms with Momordica charantia saponins and cucurbitacin B, exerting protective effects on cells and organisms through its antioxidant and immunomodulatory functions. This further supports the potential application of MV, as a cucurbitane-type triterpenoid glycoside, in protecting cryopreserved sperm during freeze–thaw processes. Overall, these results suggest that MV is a promising antioxidant for cryopreserved boar sperm, offering a theoretical foundation for improving the thawing process of frozen semen and providing technical support for preserving genetic resources in Huoshou black boars. Furthermore, this study also provides valuable insights for further research on the molecular protective mechanisms of MV on the sperm surface during the thawing process.

## 5. Conclusions

These results demonstrate that the addition of MV during the thawing process has a significant effect on improving sperm motility. This not only enhanced sperm motility and overall antioxidant levels, but also contributed to improving the efficiency of artificial insemination, which is significant for the broader application of cryopreserved pig semen.

## Figures and Tables

**Table 1:** The effects of different MV concentrations on the motility of frozen–thawed sperm.

MV (μmol/L) ^1^	Conservation Time (hours)			
	0	1	2	4
0	48.00 ± 2.95 ^a^	40.33 ± 2.28 ^a^	26.60 ± 2.24	12.90 ± 2.06
25	51.30 ± 2.17 ^ab^	44.03 ± 1.15 ^ab^	29.57 ± 2.96	11.77 ± 2.62
50	55.60 ± 2.70 ^ab^	47.07 ± 0.91 ^bc^	31.03 ± 2.89	12.53 ± 2.40
75	58.20 ± 2.63 ^bc^	49.27 ± 2.09 ^bcd^	30.33 ± 3.47	13.53 ± 2.87
100	54.20 ± 1.63 ^ab^	47.47 ± 2.14 ^bc^	32.17 ± 5.36	13.80 ± 2.26

^a,b,c,d^ Values within a column with different superscripts indicate statistically significant difference (*p* < 0.05). ^1^ 0 = not added MV; 25 = added 25 μmol/L MV; 50 = added 50 μmol/L MV; 75 = added 75 μmol/L MV; 100 = added 100 μmol/L MV.

## Data Availability

The original contributions presented in this study are included in the article. Further inquiries can be directed to the corresponding authors.
